# A Novel Tool for the Rapid and Transparent Verification of Reference Intervals in Clinical Laboratories

**DOI:** 10.3390/jcm13154397

**Published:** 2024-07-27

**Authors:** Georg Hoffmann, Sandra Klawitter, Inga Trulson, Jakob Adler, Stefan Holdenrieder, Frank Klawonn

**Affiliations:** 1 German Heart Center, Institute of Laboratory Medicine, Technical University Munich, 80636 Munich, Germany; 2 Trillium GmbH Medizinischer Fachverlag, 82284 Grafrath, Germany; 3 Institute for Information Engineering, Ostfalia University of Applied Sciences, 38302 Wolfenbüttel, Germany; 4 Institut für Hämostaseologie und Pharmakologie (IHP), 12247 Berlin, Germany; 5 Institut für Medizinische Diagnostik (IMD), 12247 Berlin, Germany; 6 Helmholtz Centre for Infection Research, 38124 Braunschweig, Germany

**Keywords:** reference interval, verification, indirect method, reflimR, refineR, color coding

## Abstract

**Background/Objectives**: We present a software package called reflimR (Version 1.0.6), which enables rapid and transparent verification of reference intervals from routine laboratory measurements. Our method makes it easy to compare the results with specified target values and facilitates the interpretation of deviations using traffic light colors. **Methods**: The algorithm includes three procedural steps: (a) definition of an appropriate distribution model, based on Bowley’s quartile skewness, (b) iterative truncation, based on a modified boxplot method to obtain the central 95% of presumably inconspicuous results, and (c) extrapolation of reference limits from a truncated normal quantile–quantile plot. **Results**: All algorithms have been combined into one consolidated library, which can be called in the R environment with a single command reflim (x). Using an example dataset included in the package, we demonstrate that our method can be applied to mixed data containing a substantial proportion of pathological values. It leads to similar results as the direct guideline approach as well as the more sophisticated indirect refineR software package. As compared to the latter, reflimR works much faster and needs smaller datasets for robust estimates. For the interpretation of the results, we present an intuitive color scheme based on tolerance ranges (permissible uncertainty of laboratory results). We show that a relatively high number of published reference limits require careful reevaluation. **Conclusions**: The reflimR package closes the gap between direct guideline methods and the more sophisticated indirect refineR method. We recommend reflimR for the rapid routine verification of large amounts of reference limits and refineR for a careful analysis of unclear or doubtful results from this check.

## 1. Introduction

Reference intervals play a crucial role in the medical interpretation and statistical evaluation of laboratory results [1]. By definition, reference intervals include the central 95% of results measured in non-diseased reference individuals [2]. It is mandatory that laboratories verify the reference limits obtained from external sources such as assay inserts or handbooks before using them for routine clinical care [2,3]. In principle, this requirement is independent of the size of the laboratory, but it is clear that small laboratories with low test numbers and higher economic pressure are more challenged here than large laboratory institutions.

In conventional approaches, the lower and upper limits of reference intervals are determined using direct methods that involve collecting laboratory results from apparently healthy individuals and calculating the 2.5th and 97.5th percentiles with parametric or non-parametric methods [2]. Although direct methods are currently considered the guideline-compliant “gold standard”, their practical application is challenging due to the cost and time issues for recruiting a sufficient number of well-defined reference individuals, as well as ethical restrictions, especially in small children, and difficulties in excluding “non-healthy” outliers [3,4,5].

This is the reason why the gold-standard procedure is only binding for the de novo definition of reference limits, while for the mere verification of otherwise defined limits, the guideline recommends a simplified approach, which just verifies that no more than two out of twenty values measured in healthy individuals fall outside the given limits [2]. However, this alternative approach is neither representative nor reproducible, nor is it able to detect reference intervals that are too wide [4].

To overcome these issues, so-called indirect methods [3] have been proposed that can be applied to larger numbers of routine laboratory results. They rely on statistical models rather than clinical measures for the definition of an apparently healthy population and attempt to derive the above percentiles from datasets containing an unknown proportion of pathological results [6,7,8,9,10,11]. The most recent of these methods has also been provided as a free software package called refineR [9], which can be downloaded from the Comprehensive R Archive Network (https://cran.r-project.org/web/packages/refineR (accessed on 2 May 2024)).

The major advantages of an R package compared to “home-brew” programs are the access via the official CRAN website, the standardized package-type documentation, and the ease of use in the R software environment. As a disadvantage of refineR, some authors, including our group, mention the relatively long computation time of the algorithm and the uncertainty of finding the right statistical model when the number of cases is below 1000 [9,10].

Therefore, we have developed an alternative R package called reflimR as refinement of our previously published, Excel-based indirect method [7]. Our main goal was to provide a much-needed tool that would allow rapid serial verification of reference intervals under routine clinical laboratory conditions [4]. To effectively support this intent, we integrated into the package an algorithm that uses traffic light colors to indicate how well the estimated reference intervals match the predefined limits used in one’s own laboratory.

In this article, we explain the functions included in reflimR, present results for the example data of the package, and compare them with those of refineR and the guideline-compliant direct method. As a special feature, the example data offer the possibility to test our method both in a direct and an indirect mode so that the influence of pathological outliers can be assessed.

## 2. Materials and Methods

All calculations and graphics were made with the free statistical software R (www.r-project.org (accessed on 2 May 2024)). Eight analytes were measured in 456 healthy controls and 156 patients with different stages of hepatitis C ranging from mild infection without histological signs to severe liver damage in the form of fibrosis and cirrhosis [12].

Table 1 exemplifies four rows from the livertests dataset included in the reflimR package. They illustrate typical values for controls and patients. The first and third rows represent a female and a male person from the healthy control group. The female patient in row 200 is an example of mild early-stage hepatitis with largely unremarkable results except for a significantly elevated GGT. The male patient in row 610, on the other hand, represents a typical cirrhotic stage with increased AST, BIL, and GGT but decreased ALB, ALT, and CHE. For the full names of the analytes see the list of abbreviations. The package also includes a list of target values (see Section 3), which were derived from the publicly available handbook of L. Thomas (https://www.clinical-laboratory-diagnostics.com (accessed on 2 May 2024)). The missing lower limits for ALT, AST, and GGT were supplemented from the manufacturer’s assay sheet.

The reflimR method falls into the category of so-called “modified Hoffmann approaches” [7,13,14], which are based on the original work of Robert G Hoffmann (1963) [15]. Their common element is that they evaluate the linear part of a regression line, which is obtained by comparing the distribution of the (eventually transformed) values with a standard normal distribution. While in the original method a probability–probability plot is generated [15], most of the newer modifications, including ours, use a normal quantile–quantile plot [7].

**Figure 1:**
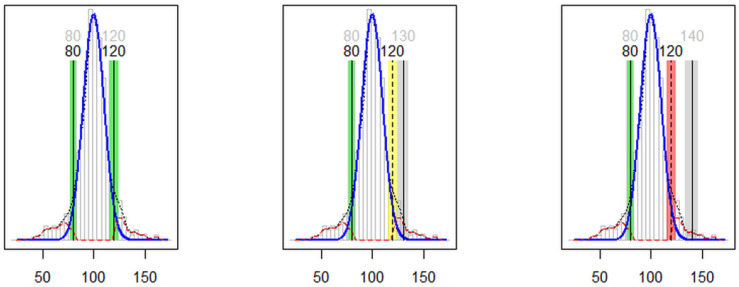
Graphical output of the reflim function. The vertical lines represent the observed and theoretical reference limits with their respective tolerance ranges.

The complete list of ten functions included in the reflimR package can be displayed with the command help (package = reflimR). The reflim function is on the highest level and represents the main function of the package. It can be called with a single command reflim (x), where x is the vector of positive numbers to be analyzed. The output of this function is a set of numeric and text results as well as a graphical representation of the calculated reference limits with colored tolerance ranges (Figure 1). The reflim function also includes a total of ten arguments with default values defining the appearance of the output. It calls the other nine functions that can be arranged as follows:

Group 1: ri_hist, permissible_uncertainty, interpretation
Group 2: lognorm, iboxplot, truncated_qqplot
Group 3: adjust_digits, bowley, conf_int95

Group 1 comprises three higher-level functions that provide the user with the final results: ri_hist creates a graphical output, permissible_uncertainty calculates the tolerance limits of the results [1,2], and interpretation assesses the medical significance of deviations from given target values. Group 2 performs the three underlying statistical operations (see Section 3), and group 3 contains auxiliary functions for miscellaneous tasks like rounding to a plausible number of digits, calculating Bowley’s quartile skewness and determining 95% confidence intervals. The details of each function are available in the respective help files, which can be addressed with a question mark followed by the function name.

Figure 1 shows an example for the graphical output of the evaluation of 1000 realistic laboratory results (e.g., blood glucose in mg/dL), simulated as three Gaussian distributions representing 80% normal values as well as 10% low and 10% high values (mean values 100, 70, and 125 and standard deviations 10, 15, and 15, respectively). The vertical lines represent the observed and theoretical reference limits. The respective tolerance ranges surrounding these vertical lines were derived from the permissible uncertainty of quantitative laboratory results [16] in a special application serving as an equivalence test for reference limits [17].

Arbitrary target values of 120, 130, and 140 were set for the upper limit in Figure 1 to illustrate the traffic light metaphor of the ReflimR approach. Green bars mean that the predicted target values lie inside the tolerance ranges of the reflim calculation. Yellow bars mean that the target values lie outside but the tolerance ranges overlap, whereas red bars mean that the tolerance ranges are completely separated. The respective interpretation outputs of the reflim function are “within tolerance” (green), “slightly increased/decreased” (yellow), and “markedly increased/decreased” (red).

A Shiny application with a graphical user interface is available to facilitate the use of reflimR for those who are not familiar with calling R functions. It can be downloaded from GitHub and installed in the R environment as described on the website (https://github.com/SandraKla/reflimR_Shiny (accessed on 2 May 2024)).

For a method comparison, the refineR package was used as a published reference [9]. This package includes two main functions that are called sequentially:

result1 <- findRI (x)
result2 <- getRI (result 1)

Briefly, the algorithm is based on the assumption that the non-pathological fraction of the data can be modeled with a Box–Cox transformed normal distribution with three parameters (mean, standard deviation, and exponent lambda). In contrast to our method, refineR starts with a sophisticated analysis of the density of the original data aiming to find a lambda value that fits a continuum of right-to-left skewed distributions rather than just our two types, i.e., Gaussian (λ = 1) and lognormal (λ = 0). In a series of complex analytical steps, the roughly transformed values are then transferred to a histogram with optimized bin width, from which a cost-based final model is obtained under various assumptions about the most likely distribution in each bin as well as in the presumably non-pathological fraction. For a more detailed description of the algorithm see Ref. [9].

Finally, we applied two direct methods to the values of the healthy control group to compare our method with the established CLSI/IFCC guideline. The “gold standard” procedure determines the 2.5th and 97.5th percentiles in healthy individuals without making any assumptions regarding the underlying distribution [2]. A simplified “20-person approach” rejects the specified reference interval if more than two out of twenty reference values fall outside its limits [2].

## 3. Results

Exploration of the livertests dataset revealed significant sex differences (*p* < 0.001 in the Wilcoxon test) but no notable age trends. Therefore, we split the data by gender into 238 women and 374 men and analyzed all age groups from 19 to 77 years collectively. The direct methods were applied to the subset of healthy blood donors, whereas the whole dataset was used for analyses with indirect methods.

**Figure 2:**
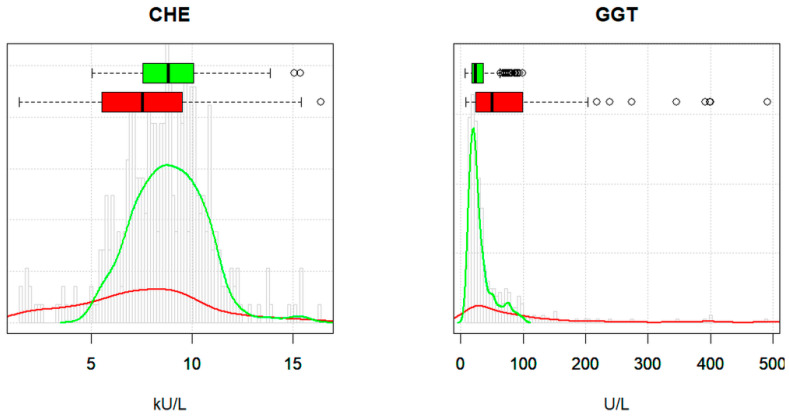
Typical density curves and boxplots for reference individuals (green) and patients (red) in the male cohort of the livertests dataset. The histogram in the background represents the distribution of all male individuals (*n* = 374).

Figure 2 shows two typical distribution curves for the reference and patient values. CHE represents an analyte with a quite symmetric distribution of the reference values, whereas the respective curve for GGT is clearly right skewed. In the patient cohort, pathological values had an accent on the left side for CHE and on the right side for GGT. In both cases, the reference and patient groups showed a large overlap, particularly at the critical border between normal and pathological values. Estimates of the percentage of non-pathological results in the mixed data ranged from 82 to 99% (median 90%) for reflimR and from 74 to 97% (median 88%) for refineR. Both figures are higher than the real percentage of healthy controls in our study group (75%). This seeming discrepancy is due to the high amounts of “normal” results in the patient group.

### 3.1. Step-by-Step Explanation of the reflimR Algorithm

**Figure 3:**
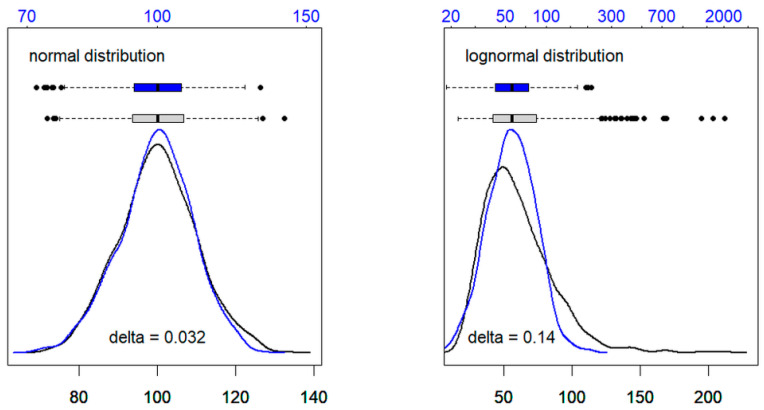
Graphical output of the lognorm function (step 1 of the reflim algorithm). The graphics represent density curves and boxplots of 1000 simulated values with arbitrary units (black: original, blue: logarithms). The **left side** shows normally distributed values (mean = 100, sd = 10), whereas the **right side** is an example of a lognormal distribution (meanlog = 4.0, sdlog = 0.4). For the calculation of Bowley’s skewness delta see Table 2.

**Figure 4:**
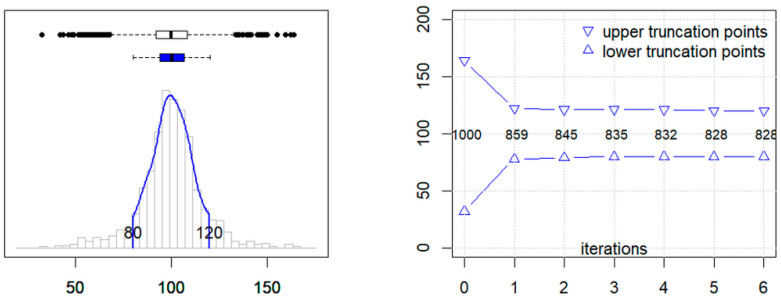
**Left graphic**: histogram of the original values with the corresponding boxplot in black. The blue curve represents the density of the truncated values with corresponding boxplot in blue. **Right graphic**: number of values remaining after each step of the iterative truncation.

**Figure 5:**
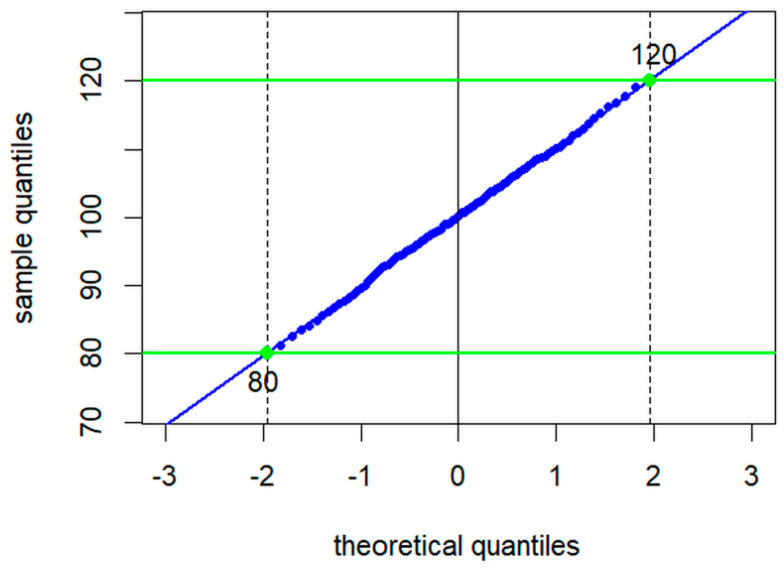
Graphical output of the truncated Q-Q plot function.

Figure 3, Figure 4 and Figure 5 illustrate the three main steps performed by the reflim function. The algorithm includes three procedural steps: (a) definition of an appropriate distribution model, based on Bowley’s quartile skewness, (b) iterative truncation, based on a modified boxplot method to obtain the central 95% of presumably inconspicuous results, and (c) extrapolation of reference limits from a truncated normal quantile–quantile plot.

**Table 2:** Bowley’s skewness calculated with the lognorm function for the normally and lognormally distributed values shown in Figure 3.

	Normal Distribution	Lognormal Distribution
original values	0.014	0.133
logarithms	−0.018	−0.007
delta ^1^	0.032	0.140

^1^ delta is the difference of the calculated Bowley’s skewness of the respective original values and their logarithms.

In step 1 (Figure 3), a suitable distribution model is defined by calculating Bowley’s quartile skewness coefficients (i.e., the skewness of the central 50% of all values) for the measured values and their logarithms. In short, this algorithm (called lognorm) checks whether the difference of the two skewness coefficients exceeds an empirical threshold of 0.05 [18]. On the left side of Figure 3, both the black and the blue curves are about equally symmetric, so that their skewness delta is below 0.05. On the right side, the black curve is clearly right skewed and the blue density curve of the logarithms is much more symmetric. Due to this marked difference in shape, the delta is greater than 0.05.

The rationale for this algorithm is that analytes in blood can be roughly divided into two physiologically defined classes [1], one of which has physiological functions in blood and is therefore tightly regulated (e.g., albumin, hemoglobin, sodium). The values of this group show little variation. The second group has no specific function in blood (e.g., AST, ALT) and therefore varies widely. The distribution of the first group is quite symmetric (skewness ≈ 0), so that the Gaussian distribution model fits them well within the range of the assumed reference limits. The distribution of the second group is usually right skewed (skewness >> 0) and becomes substantially more symmetric after log transformation (skewness difference > threshold). In this case, a lognormal distribution should be assumed, which becomes normal after a log transformation of the data [18,19].

In step 2, the (possibly transformed) dataset is truncated in an iterative way such that the presumably pathological values are removed as best as possible and the central 95% of the presumably non-pathological values remain without substantial losses. To achieve this, we adopt the iboxplot algorithm [18], which is based on Tukey’s boxplot method. Starting with the central 50 percent of the data (i.e., the first and third quartiles), the theoretical 2.5th and 97.5th percentiles of a Gaussian distribution are calculated and values beyond these limits are removed. This algorithm is repeated until the length of the vector remains constant, i.e., no more values are removed. After the first truncation step, the calculation of the 2.5th and 97.5th percentiles is adapted to the fact that the vector has already been truncated. For more details, we refer to the original publication on the iBoxplot95 algorithm [20].

Figure 4 illustrates the outcome of the step 2 iboxplot function for the same data as in Figure 1. The left graphic shows the histogram of the original values with the corresponding boxplot (white). The blue curve represents the density of the truncated values with the corresponding boxplot in blue. The majority of the pathological values are removed in the first truncation step (drop from *n* = 1000 to *n* = 859) (Figure 4, right). The following iterations very slowly approach the target interval of 80 to 120, which is reached at a total number of *n* = 828.

In the third and final step (Figure 5), a normal quantile–quantile plot (Q-Q plot) is generated as described earlier [7], with an important modification: in the original version, the linear part of the curve was identified visually and then replotted against the quantiles of a standard normal distribution, whereas in the present package version, the truncated_qqplot function plots the quantiles of the truncated vector against the respective quantiles of a standard normal distribution truncated between the 2.5th and 97.5th percentiles. A total of 39 quantiles between equidistant probabilities from *p* = 0 to *p* = 1 are calculated from the truncated sample and plotted against 39 quantiles of a standard normal distribution with equidistant probabilities between *p* = 0.025 and *p* = 0.975. This function determines the mean and standard deviation from the intercept and slope of the regression line and extrapolates the reference limits from mean ± 1.96 sd.

### 3.2. Verification of Reference Limits and Method Comparison

**Figure 6:**
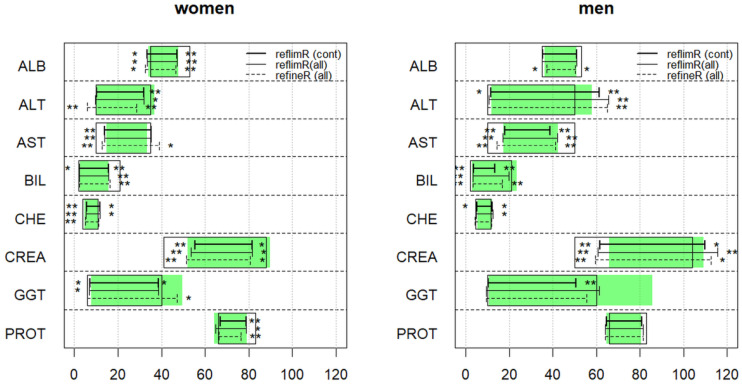
Comparison of results obtained with reflimR (solid lines) and refineR (dashed line). The reflimR method is applied to healthy controls (thick line) and to all individuals (thin line). The green boxes indicate the target values determined with the direct quantile-based CLSI/IFCC method applied to the healthy controls, and the empty rectangles represent the target values derived from the literature. * = slight deviation from the literature. ** = marked deviation from the literature. The terms slight and marked refer to the yellow and red traffic light colors shown in Figure 1. For analyte-specific units on the x-axis, see Table 2.

The reflimR algorithm, which calls these three functions consecutively, was tested both in a direct and in an indirect mode (see Section 2). For a comprehensive method comparison, we also applied the two direct guideline methods and the indirect refineR method. The results are summarized in Figure 6 and in Table 3.

The figure provides a concise visualization of the results obtained with reflimR (horizontal solid lines) performed either on the healthy controls or on the whole dataset. Except for 3 out of 32 reference limits (i.e., upper limits for BIL, CREA, and GGT in men), the results are in excellent agreement, confirming that under typical routine conditions our indirect method is largely unaffected by pathological values. A similarly good agreement is achieved with the results of refineR (horizontal dashed lines), whereby the latter method performs slightly better for the three exceptions mentioned above.

In addition, Figure 6 compares the results of all three methods with target values calculated with the direct “gold standard method” (green boxes) and with those derived from the literature (empty boxes). The degree of deviation from the literature values is indicated by one or two asterisks for “slight” or “marked” discrepancies, which correspond exactly to the yellow and red colors, respectively, in the reflim function. No systematic differences are observed here between the two indirect methods (reflimR and refineR): sometimes both result in reference ranges that are wider than expected from the boxes (e.g., AST in women and ALT in men) and sometimes both yield narrower intervals (e.g., BIL and CREA in women).

In essence, the differences between the two indirect methods (solid versus dashed lines) are much smaller than the discrepancies among the corresponding intervals obtained from the gold-standard method and the literature (green versus empty boxes). Most remarkably, the gold standard is not beyond all doubt either. For example, here the upper limit for GGT in men appears to be significantly too high. The reason becomes apparent from Figure 2, where the GGT values measured in the apparently healthy reference collective include several moderately elevated values (e.g., due to undocumented alcohol or drug consumption), which may contribute to a 97.5th percentile that is too high.

This brings us to the final and crucial question of this work: does the reflimR method fulfill the goal formulated at the beginning of this article, namely, to provide an instrument that enables reference intervals from external sources to be verified quickly on the basis of easily interpretable traffic light colors? For this purpose, we transformed the results depicted in Figure 6 into ordinal decisions “accept” (green = within tolerance), “check” (yellow = slightly increase/decreased), and “reject” (red = markedly increase/decreased). Then, we compared these categories with the “20-person” verification method described in the CLSI/IFCC guideline [2]. Table 2 shows that reflimR rejects 11 of 32 reference limits and suggests another 7 for review, whereas the guideline method does not differentiate between exceeding the lower or upper reference limits and accepts almost all reference intervals from the literature (see discussion). In our experiment, it rejects only 0 to 2 out of 16 reference intervals both in women and men. This means that our method assesses the specified reference intervals much more strictly than the simplified guideline method.

An important side finding from our method comparison is the enormous difference in computing times under the conditions of the livertests dataset (Figure 6): for reflimR, the time was 0.006 to 0.017 s, whereas for refineR, the corresponding time was 47 to 99 s. On average, reflimR was more than 6000 times faster than refineR.

**Figure 7:**
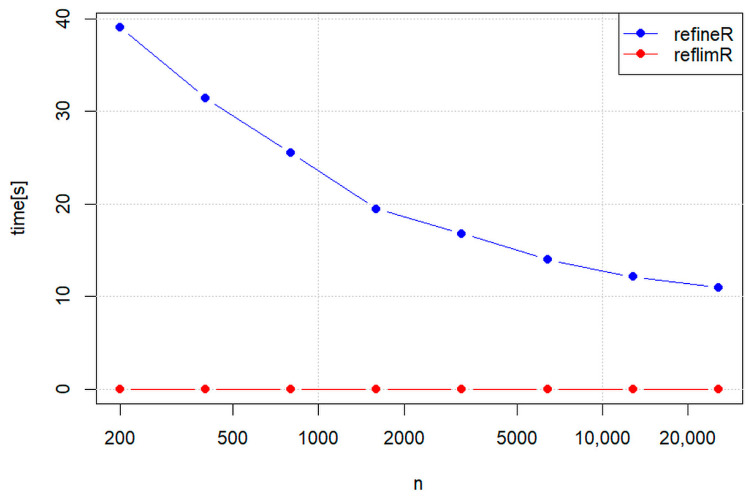
Computation times of reflimR and refineR as a function of sample sizes. Each point represents the mean of 30 simulations of normally distributed values without outliers.

To examine whether this statement can be generalized, we tested the computation times as a function of the number of observations using a very simple normally distributed dataset. Figure 7 shows that with increasing size, reflimR becomes slightly slower, whereas refineR becomes remarkably faster. Nevertheless, even at *n* > 20,000, reflimR was more than 3000 times faster than refineR in this example.

## 4. Discussion

The indirect reflimR method presented here is suitable for a quick and easy-to-interpret verification of specified reference intervals. It works well with mixed datasets containing up to 25% patients with confirmed disease. Taking the livertests dataset included in the package as example, reflimR accepts less than 50% of the literature-derived limits: 34% are rejected and 22% are classified as worth reviewing.

The color system presented here makes it easier to quickly assess the agreement of the reflimR results with reference limits from handbooks or assay package inserts. The traffic light colors are intuitive but not subjective, as they are determined by the specifications of the permissible uncertainty [16,17] and cannot be influenced by the user. In contrast to statistical confidence intervals [2], the permissible uncertainty is independent of the number of observations. The confidence intervals provided by reflimR are a valuable reproducibility measure in cases with low numbers of values but become extremely narrow when several thousand values are analyzed.

If we take a closer look at the red and yellow fields in Table 3, it is noticeable that more limits are affected in women than in men. Among them are ALB and BIL, where the target values make no difference between both genders, whereas our data indicate a significant difference in the medians (*p* < 0.001). A brief literature search shows that men do indeed have higher albumin and bilirubin concentrations than women [21,22], a fact that is rarely considered by assay manufacturers and clinical laboratories.

The results of our method are similar to those of the direct CLSI/IFCC method [2] as well as the more complex refineR method [9]. No notable differences are observed when our method is applied to the healthy controls only, i.e., omitting the patient values. In this latter case, reflimR and refineR may even outperform the so-called gold standard in specific situations, i.e., where the healthy control group contains individuals with slightly pathological results (see the green density curve in Figure 2 and the green boxes for GGT in Figure 6). Such borderline values are reliably eliminated by the three-stage procedure used here, whereas they are fully reflected in the results when calculating quantiles alone without any model assumptions.

With older methods [6,15,23], an IFCC working group found that the application of indirect methods to mixed populations resulted in some bias as compared to carefully selected healthy reference individuals [24]. This limitation may also apply to reflimR and refineR (see for example CREA for men in Figure 6), but does not seem to be too serious if these methods are just used for verification of already existing reference intervals rather than for establishing them de novo.

Compared to refineR, the much higher speed is an outstanding advantage of our method. Figure 7 shows that the computation time of refineR decreases as a function of the number of observations. This counterintuitive behavior can probably be explained by the complex statistical algorithm, which leads to faster convergence for larger sample sizes [9]. Nevertheless, reflimR is several thousand times faster and therefore qualifies for the rapid verification of reference limits. The long calculation times of refineR may not play a role in individual analyses but can quickly become a problem if the algorithm has to be run repeatedly.

This is particularly the case when confidence intervals are calculated with simulation or bootstrap techniques [2,25]. The conf_int95 function of reflimR is based on 100,000 Monte Carlo simulations for each sample size from 200, 400, 600 … to 2000 (see conf_int95 in the package documentation). While this experiment with a total of one million simulations takes about two hours, the corresponding duration with refineR would be roughly a year on the same computer. Very long computation times may also be a reason why refineR does not output any confidence intervals in the default setting. The documentation only contains very rough calculation examples with 30 bootstraps that already take several minutes. For a standard lognormal distribution with 10,000 values, refineR returns plausible reference limits of 0.13 (CI95 0.10 to 0.14) and 6.94 (CI95 4.50 to 7.21) after about three minutes. The same simulation performed with reflimR takes 50 milliseconds and yields almost identical reference limits of 0.14 (CI95 0.10 to 0.19) and 6.88 (CI95 6.05 to 7.70). The reflimR algorithm is so fast because here the confidence intervals are calculated with closed formulas that are based on the 100,000 simulations mentioned above. Such formulas do not exist for refineR.

Several publications have dealt with the minimum sample size required for the different methods. Due to the specifications in the guideline [2], it has become common practice to consider a sample size of 120 values as a minimum [9,25,26]. A critical IFCC document published in 2010 states that this number is far from optimal and at least 400 healthy individuals are desirable [27]. The reflimR algorithm issues a warning if there are less than 200 presumably inconspicuous values remaining after truncation of the original data. For a very clean sample without pathological outliers, reflimR even works with only 40 values, from which the 39 quantiles of the Q-Q plot can be calculated (see step 3 visualized in Figure 5). This number is considered the absolute minimum for the reflimR method if performed in a direct mode with healthy subjects only.

In contrast, refineR warns if there are less than 1000 values in the total dataset. The latter figure has been confirmed by Anker et al. [10], who found that reflimR is unable to estimate plausible lambda values at sample sizes below 1000. Our results, summarized in Figure 6, suggest, however, that there are no notable differences between reflimR and refineR even for sample sizes below 1000.

Finally, and most importantly, our method is clearly superior to the simplified guideline approach, which uses just twenty values from healthy controls and counts how many of them fall outside the specified reference interval [2,4,26]. The poor reproducibility of the results (see Table 3) shows that twenty individuals are not enough for a representative sample of the healthy population and, in addition, the simplified guideline method is inherently flawed because it cannot recognize reference intervals that are too wide [4]. In our study, this severe limitation applies to ALB and BIL in women and AST in men, as well as CREA in both genders (see empty boxes in Figure 6). On the other hand, the guideline method tends to reject reference intervals erroneously if the seemingly healthy population includes sick individuals with slightly pathological values (see GGT for men).

### Limitations of the Study and Outlook

As a rule, the results of reflimR do not differ significantly from those of refineR and it makes no difference whether reflimR is applied to data from healthy individuals or to mixed populations (see Figure 6). Slight deviations from this rule, such as for GGT or CREA for men, are probably due to an accumulation of borderline pathological values, which are difficult to separate from the values of healthy individuals using our algorithm (Figure 2). Such borderline cases are identified by visual inspection of the quantile–quantile plot (Figure 5). Deviations from linearity indicate the need for analyses using methods like refineR as part of quality assessment. However, objective criteria for non-linearity are still lacking [7,10].

Other questions that can be addressed with real-world data include handling small amounts of data in specialty testing [10], determining the lower reference limits below the detection level of an assay [8], or integrating reflimR and refineR into laboratory information systems.

Noteworthily, it was demonstrated that reflimR in its indirect version produces results comparable to more sophisticated direct methods and the more time-consuming refineR method. Previous studies have shown similar concordance for the precursor methods of reflimR [10,11]. Independent multi-center studies with real laboratory data are needed to validate the performance of reflimR under all conceivable routine conditions and to define when other methods such as refineR need to be used as a control. This also applies to the question of whether the high rate of rejections of predefined reference intervals (see Table 3) by reflimR is also confirmed by other methods.

The general applicability of indirect methods for testing reference intervals is still a matter of debate [3,24,27], but as direct methods also have their limitations (see Figure 6 and Table 3), clear criteria for the use of healthy reference subjects versus mixed populations need to be defined. We hope that our simple, intuitive, and fast method will pave the way for comprehensive investigations and foster collaboration between scientists in laboratory medicine and statistics.

## 5. Conclusions

In conclusion, we recommend reflimR for the rapid, routine verification of large numbers of reference limits and refineR for a careful analysis of unclear results of this examination. If the results from the two methods are doubtful or do not coincide, a direct approach should be considered. The simplified direct guideline method should, however, no longer be used for verifying external sources, as it accepts too many false reference intervals (Table 3) and lulls the user into a false sense of security.

## Figures and Tables

**Table 1:** Excerpt from the livertests dataset included in the reflimR package.

Row	Category	Age	Sex	ALB	ALT	AST	BIL	CHE	CREA	GGT	PROT
1	reference	32	f	39.9	22.0	29.8	6.3	8.16	60	4.5	72.5
200	patient	51	f	41.4	33.2	20.0	5.0	10.27	77.0	106.7	72.2
444	reference	54	m	46.4	54.1	39.6	10.6	6.59	85	73.2	75.2
610	patient	59	m	31.0	5.4	95.4	117.0	1.57	60.5	53.6	68.5

**Table 3:** Target reference intervals and verification results obtained from two verification methods. a = accept (green), c = check (yellow), r = reject (red), f = female, m = male, ll = lower limit, ul = upper limit. reflimR was applied to the whole dataset, whereas for the guideline method, three random samples with *n* = 20 each (f1, 2, 3 and m1, 2, 3) were drawn from the healthy control group.

	Reference Interval		reflimR				Guideline Method					
	f	m	ll f	ul f	ll m	ul m	f1	f2	f3	m1	m2	m3
ALB (g/L)	35–53	35–53	c	r	a	a	a	r	a	a	a	a
ALT (U/L)	10–35	10–50	a	c	a	r	r	a	a	a	r	a
AST (U/L)	10–35	10–50	r	a	r	r	a	a	a	a	a	a
BIL (µmol/L)	2–21	2–21	a	r	r	a	a	a	a	a	a	a
CHE (kU/L)	3.9–10.8	4.6–11.5	r	c	a	c	a	a	a	a	a	a
CREA (µmol/L)	41–88	50–104	r	c	r	r	a	a	a	a	a	a
GGT (U/L)	6–40	10–60	c	a	a	a	a	r	a	a	r	r
PROT (g/L)	66–83	66–83	a	c	a	a	a	a	a	a	a	r

## Data Availability

The data set used in this study comes along with the R package reflimR.

## Abbreviations

ALB	albumin
ALT	alanine aminotransferase (GPT)
AST	aspartate aminotransferase (GOT)
BIL	bilirubin
CHE	choline esterase
CLSI	Clinical Laboratory Standards Institute
CREA	creatinine
GGT	gamma-glutamyl transferase
IFCC	International Federation of Clinical Chemistry and Laboratory Medicine
PROT	total protein
sd	standard deviation
